# In Situ Investigation of Microstructural Evolution and Intermetallic Compounds Formation at Liquid Al/Solid Cu Interface by Synchrotron X-ray Radiography

**DOI:** 10.3390/ma15165647

**Published:** 2022-08-17

**Authors:** Fei Cao, Ruosi Wang, Peng Zhang, Tongmin Wang, Kexing Song

**Affiliations:** 1 Shaanxi Province Key Laboratory for Electrical Materials and Infiltration Technology, School of Materials Science and Engineering, Xi’an University of Technology, Xi’an 710048, China; 2 School of Materials Science and Engineering, Dalian University of Technology, Dalian 116024, China; 3 School of Materials Science and Engineering, Henan University of Science and Technology, Luoyang 471023, China

**Keywords:** liquid Al/solid Cu interface, bubble growth, intermetallic compounds, microstructure, synchrotron X-ray radiography

## Abstract

Synchrotron radiation dynamic imaging technology combined with the static characterization method was used to study the microstructural evolution and the growth kinetics of intermetallic compounds (IMCs) at the liquid Al/solid Cu interface. The results show that the interfacial microstructure can be divided into layered solid diffusion microstructures (AlCu_3_, Al_4_Cu_9_, Al_2_Cu_3_ and AlCu) and solidification microstructures (Al_3_Cu_4_, AlCu and Al_2_Cu) from the Cu side to the Al side. Meanwhile, the growth of bubbles formed during the melting, holding and solidification of an Al/Cu sample was also discussed, which can be divided into three modes: diffusion, coalescence and engulfment. Moreover, the growth of AlCu_3_ and (Al_4_Cu_9_ + Al_2_Cu_3_) near the Cu side is all controlled by both interfacial reaction and volume diffusion. The growth of Al_3_Cu_4_ adjacent to the melt is mainly controlled by the interfacial reaction, which plays a major role in the growth of the total IMCs.

## 1. Introduction

Al/Cu bimetals are an important type of composite material combining the high corrosion resistance, lightweight, economic and aesthetic properties of aluminum with the low contact resistance and high thermal and electrical conductivity of copper [1,2]. This type of material has been widely applied in many industrial fields, such as the automobile, power communication, electrics and electronics fields [3,4].

At present, there are many methods to prepare Al/Cu bimetals, such as diffusion bonding [5], rolling bonding [6], transient liquid phase bonding [7] and continuous casting [8]. Among all the above-mentioned methods, continuous casting is a promising technique with good metallurgical bonding, low cost and high efficiency. Research [9,10] has shown that the formation of interfacial microstructure plays a key role in the interface control and properties of bimetals. Therefore, to obtain a better understanding of the liquid–solid reaction involved in the compound process is technically and scientifically important for the production of Al/Cu bimetal with a good metallurgical bonding interface by continuous casting. Previous studies [11,12] have shown that liquid–solid bonding is mainly realized through the combined effect of local melting of the interface (fusion bonding mechanism), interatomic diffusion and diffusion reaction (diffusion bonding mechanism). However, it lacks dynamic observation of non-equilibrium solidification of interfacial melt and the growth of intermetallic compounds (IMCs), which need in situ characterization for further study.

However, due to the lack of real-time in situ observation methods and the opacity and high temperature characteristics of the metals, the dynamic observation of the interfacial microstructure evolution is limited to a large extent. Thus, the interfacial bonding mechanism is usually inferred based on a large number of static experiments [13,14], lacking direct experimental evidence. With the development of third-generation high performance synchrotron radiation facilities, the synchrotron radiation imaging technique has been applied and developed unprecedentedly in the field of metallic materials [15,16,17]. This imaging technique can also be applied to uncover the interfacial microstructure evolution of bimetals from the perspective of micro-nano scale and dynamics.

In this study, we employ synchrotron radiation imaging technology and focus on the microstructural evolution and IMC formation during the melting, holding and solidification of an Al/Cu sample. Interfacial microstructure evolution and phase determination are examined. The growth kinetics of IMCs are determined simultaneously. Moreover, the growth of bubbles is also discussed based on the in situ observation. This work explores an avenue for regulating interfacial microstructure and optimizing the interfacial bonding of Al/Cu bimetals prepared by the liquid–solid compound method.

## 2. Materials and Methods

**Figure 1:**
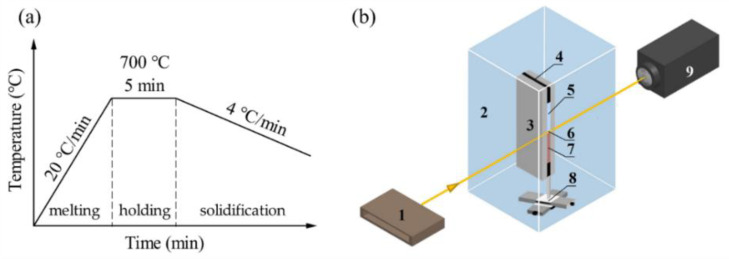
(**a**) The schematic of the heating and cooling profile; (**b**) the schematic diagram of synchrotron radiation experimental setting. 1 Synchrotron radiation X-ray, 2 vacuum furnace, 3 ceramic plate, 4 mica sheet, 5 pure Al, 6 interface, 7 pure Cu, 8 sample holder, 9 CCD camera.

Pure Al plate (99.99 wt.%) and pure Cu plate (99.97 wt.%) were mechanically compounded by cold rolling to prepare Al/Cu diffusion couples. Firstly, the Al/Cu samples were carefully ground and fine polished into thin samples with a 10 × 4 mm^2^ surface area and a 200 μm thickness, respectively. Secondly, the thin Al/Cu sample was placed in a hollow mica sheet, then further clamped by two ceramic plates and fixed with a molybdenum clamp. Finally, the assembled Al/Cu sample was placed in a self-designed vacuum furnace and heated to 700 °C with a heating rate of 20 °C/min. At this temperature, the Al was melted but the Cu was still solid. After the sample was held at 700 °C for 5 min, the solidification experiment was carried out. The cooling rate was kept at 4 °C/min. The schematic of the heating and cooling profile is shown in Figure 1a.

The experiments were carried out on beamline BL13W1 of the Shanghai Synchrotron Radiation Facility (SSRF) in Shanghai, China, using a monochromatic 22 keV X-ray beam. A YAG: Ce scintillator screen was used to convert the transmitted X-rays to visible light. The time-sequenced images of the microstructural evolution and IMC formation at the liquid Al/solid Cu interface were recorded by a fast-read-out, low-noise charged couple device (CCD, Hamamatsu, Japan) camera with a resolution of 0.65 μm per pixel at a frequency of 1 frame per second. The radiography image quality is improved by image processing [18], which consists of subtracting the original image of the sample taken at a time t by a reference image recorded just before the experiments. The schematic diagram of the synchrotron radiation experimental setting is shown in Figure 1b.

Scanning electron microscopy (SEM, Zeiss Supra 55, Carl Zeiss, Germany) was carried out post-mortem after the experiment at SSRF to characterize the interfacial microstructure of the Al/Cu sample. Combined with the Al–Cu binary phase diagram, the type of IMCs in the diffusion zone was examined using energy dispersive spectroscopy (EDS).

## 3. Results and Discussion

### 3.1. Dynamic Evolution of Diffusion and Solidification

**Figure 2:**
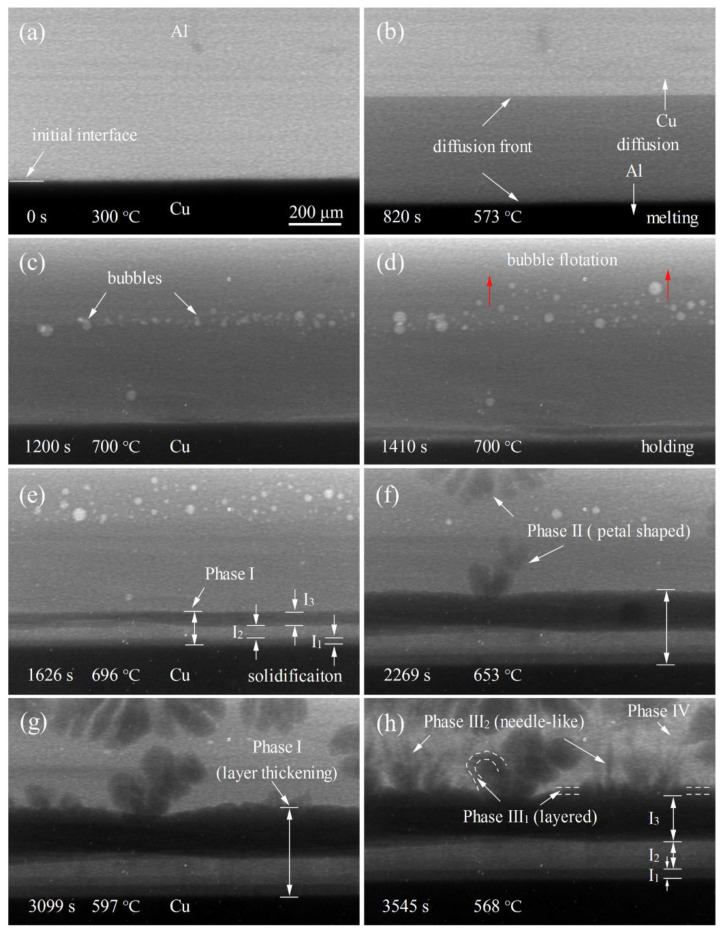
Sequence of in situ radiographs showing the interfacial diffusion and microstructure evolution in the Al/Cu sample: (**a**–**d**) the melting and holding process (*t* = 0 s is assigned to the onset of image collection at 300 °C), (**e**–**h**) the solidification process after holding at 700 °C for 5 min.

Figure 2 shows a sequence of in situ radiographs of the interfacial diffusion and microstructure evolution of the Al/Cu sample during melting, holding and solidification. The bright area on the upper side is the Al sample, and the dark area on the lower side is the Cu sample.

Figure 2a–d shows the melting and holding process of the Al/Cu sample. The Al and Cu elements diffused mutually at the initial interface of the Al/Cu sample and formed a clear straight diffusion front (Figure 2b), indicating that the initial interface of the Al/Cu sample has good mechanical bonding. With the increase in temperature, the diffusion fronts are gradually moving forward, resulting in an increasing concentration of Cu in the Al side sample and Al in the Cu side sample. When the temperature rises to 644 °C, bubbles begin to form on the Al side sample, as shown in Figure 2c. The formation and movement of bubbles, which will be discussed in Section 3.2, indicate that the Al side sample is already in a liquid state. According to the Al-Cu phase diagram, an Al–Cu alloy with a Cu concentration in the range of 8–58 wt.% will be liquid at this temperature, indicating that diffusion dissolution occurs in the Al/Cu sample. 

Figure 2e–h show the solidification process of the Al/Cu sample after holding at 700 °C for 5 min. Based on the mutual diffusion of the Al and Cu elements in the early stage, phase I near the Cu side began to grow in a layered manner and gradually thickened. According to the contrast of images, phase I can be roughly divided into three layers (I_1_, I_2_ and I_3_), as shown in Figure 2e. It should be noted that the brightest area should be I_3_ when considering the composition in Cu, I_1_, I_2_ and I_3_ layers, but it appears in I_2_. It might be because the thickness of the I_3_ layer, which is near the liquid phase, is thicker than that of the I_2_ layer, resulting in less X-ray transmissiveness. With the decrease in temperature, phase II begins to form and grow on the surface of phase I_3_ and in the melt in front of it, presenting a petal-like morphology (Figure 2f), while phase I continues to grow in a layered manner during this process (Figure 2g). At the later stage of solidification, phase III begins to grow on or near the surface of phase I_3_ and phase II, and its morphology can be divided into layered (III_1_) and needle-like (III_2_), as shown in Figure 2h. Finally, phase IV (the bright area in Figure 2h) formed at the end of solidification. The phase determination, interfacial microstructure formation and the growth kinetics of IMCs will be discussed in Section 3.3 and Section 3.4.

### 3.2. Formation and Evolution of Bubbles

The gas evolution during melting, holding and solidification always resulted in the formation of porosities in the liquid/solid interconnection, which was detrimental to electrical conductivity and mechanical properties [19]. Therefore, it is vital to understand the formation and growth behavior of the bubbles in order to achieve porosity-free products [20]. Electrical and Mechanical Performance.

**Figure 3:**
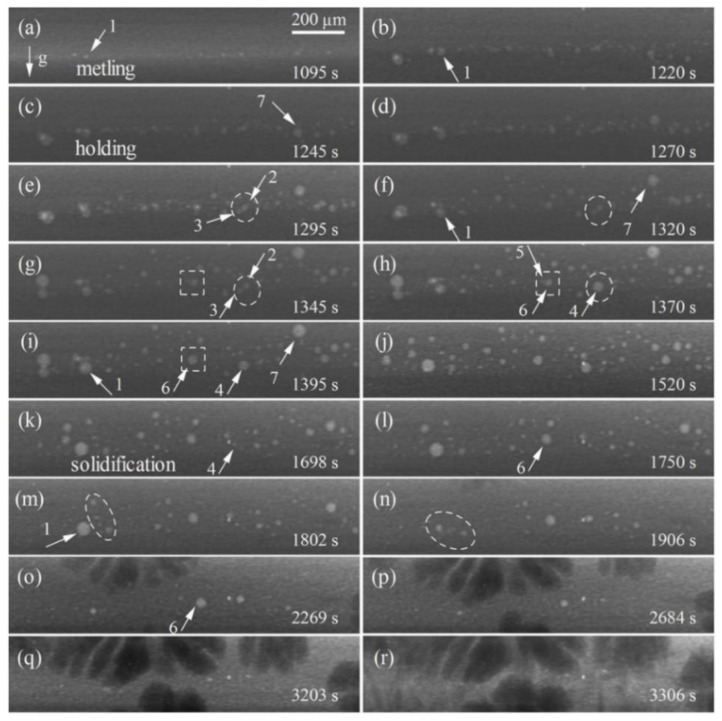
The formation and evolution of bubbles: (**a**,**b**) the melting process (*t* = 0 s is assigned to the onset of image collection at 300 °C), (**c**–**j**) the holding process, (**k**–**r**) the solidification process.

Figure 3 shows the formation and evolution of bubbles during the melting, holding and solidification of the Al/Cu sample. The sequence of in situ radiographs shows that the bubbles formed and grew on the Al side. Meanwhile, the coalescence, engulfment and floating up of bubbles were also observed. Finally, the bubbles gradually collapsed and disappeared. In order to further study the evolution of bubbles in-depth, several representative bubbles (No. 1–7 in Figure 3) were selected for detailed analysis.

*Bubble nucleation*: Due to the rapid nucleation of bubbles and the limited time/spatial resolution of the synchrotron radiation imaging method, the bubble nucleation is difficult to directly observe. Classical nucleation theory is usually used to analyze the nucleation of bubbles. Research [21,22,23] shows that bubbles tend to nucleate on the surface of oxide inclusions in the melt. In the case of heterogeneous nucleation, the total free energy change of the system during bubble nucleation can be expressed as:

(1) $$\Delta G_{het}=\Delta G_{hom}f\left(\theta \right)$$

where, *f* (*θ*) is the shape factor, which is closely related to the contact angle *θ*, and can be expressed as:

(2) $$f\left(\theta \right)=\frac{2-3cos\theta +cos^{3}\theta }{4}$$

According to Equations (1) and (2), the bubbles are more likely to heterogeneous nucleation on the oxide surface when the concentration of gas in the melt gathers to a certain extent.

**Figure 4:**
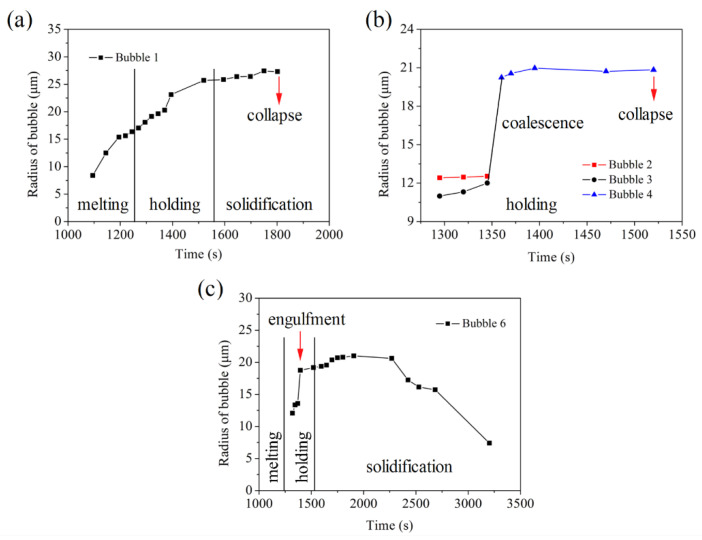
The variation of the radius of bubbles with time. Bubbles grow up through (**a**) diffusion, (**b**) coalescence and (**c**) engulfment.

*Bubble growth*: Figure 4 shows the variation of the radius of bubbles with time. The bubble growth can be divided into three modes: diffusion, coalescence and engulfment, which are similar to the growth mode of the second phase droplet during the liquid–liquid phase separation of the immiscible alloy [24]. Growth mode 1: The radius of bubble 1 gradually increases with time and stabilizes at 26.5 μm, as shown in Figure 4a, which is realized by its continuous absorption of supersaturated gas in the melt. Finally, bubble 1 collapsed and disappeared, leading to an obvious perturbation of the melt and promoting the surrounding bubbles to move to the position of the ruptured bubble 1 (elliptic region in Figure 3m,n). Growth mode 2: Bubble coalescence, which usually occurs between two bubbles with similar radius. The bubble 2 (12.5 μm) and bubble 3 (11.4 μm), with a similar radius, coalesced to form a larger bubble 4 (20.7 μm) in the holding stage (circular region in Figure 3e–h), as shown in Figure 4b. Then, bubble 4 stopped growing and eventually collapsed and disappeared. Growth mode 3: Bubble engulfment occurs between two bubbles with a large difference in radius. The radius of bubble 6 suddenly increases from 13 μm to 19 μm by engulfing the smaller bubble 5 around it (rectangular region in Figure 3g–i) and stabilizes at 20 μm, then the radius of bubble 6 gradually decreased and finally disappeared.

*Bubble floating*: The floating up of bubble 7 is also observed at the initial stage of growth, as shown in Figure 3f–i. The bubble is lighter compared to the melt. Thus, the density difference provokes the floating up of the bubble. During the floating process, the bubble was affected by gravity, buoyancy and viscous resistance of the melt, and finally reached equilibrium and stopped floating.

### 3.3. Determination and Evolution of Interfacial Microstructure

**Figure 5:**
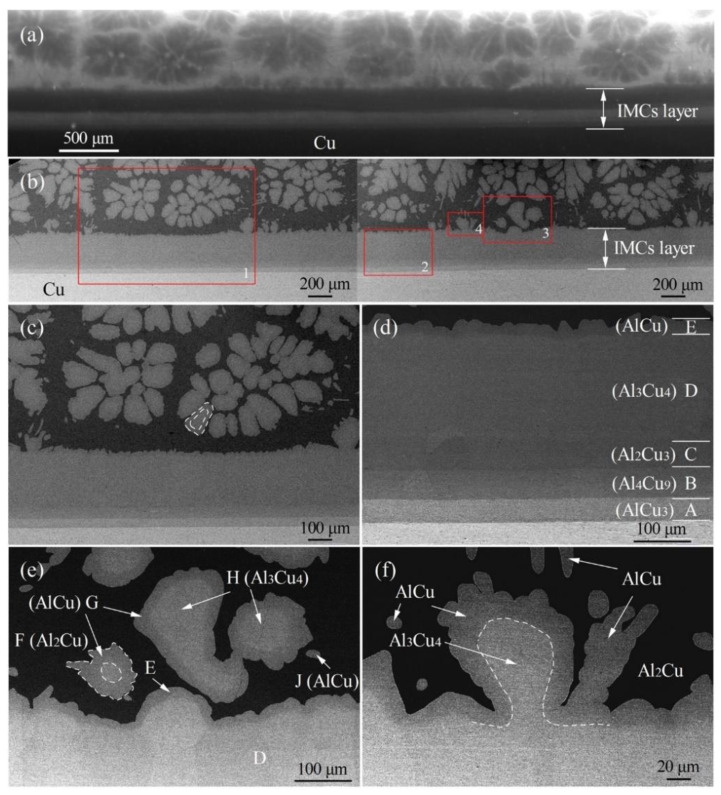
Characterization of interfacial microstructure in Al/Cu sample: (**a**) in situ radiograph, (**b**) SEM image corresponding to Figure 5a, (**c**–**f**) enlarged images of red rectangular areas in Figure 5b.

**Figure 6:**
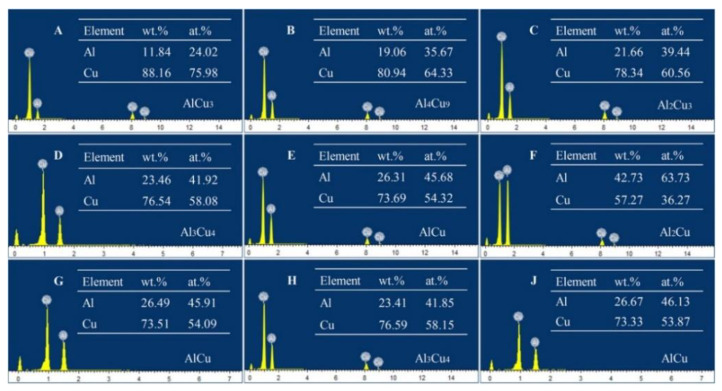
Composition analysis of interfacial microstructures (the phases (**A**–**J**) in Figure 5d,e) in Al/Cu sample by EDS.

In order to further analyze the formation of interfacial microstructures, the morphology characterization in Al/Cu samples after the synchrotron radiation imaging experiment was carried out by SEM, as shown in Figure 5. Moreover, based on the EDS composition analysis and combined with the Al–Cu phase diagram, the phases A–J in Figure 5d,e were also determined, as shown in Figure 6. Based on the in situ dynamic imaging (Figure 2) and static characterization (Figure 5), the phases and morphology of the interfacial microstructure were finally determined, as shown in Table 1. The formation of the interfacial microstructure of Al/Cu samples is analyzed as follows.

At the initial stage of solidification, the phases I_1_ (A: AlCu_3_), I_2_ (B + C: Al_4_Cu_9_ + Al_2_Cu_3_) and I_3_ (D: Al_3_Cu_4_) near the Cu side first grew in a layered manner and gradually thickened, with the final lamellar thickness of 40 μm, 104.5 μm and 175.2 μm, respectively, as shown in Figure 2e–h and Figure 5d. With the decrease in temperature, the petal-shaped phase II (H: Al_3_Cu_4_) grows independently in the melt or attached to the layered phase I_3_ (Al_3_Cu_4_), as shown in Figure 2f,g and Figure 5e,f. Both phase I_3_ and phase II with different morphologies are Al_3_Cu_4_, indicating that they have different growth modes. The lamellar Al_3_Cu_4_ mainly grows in diffusion mode, which will be analyzed in detail in Section 3.4. While the petal-shaped primary Al_3_Cu_4_ mainly formed through solidification (L → Al_3_Cu_4_). After that, phase III_1_ (E and G: AlCu) begins to grow on the surface of the layered and petal-shaped Al_3_Cu_4_ through peritectic reaction (L + Al_3_Cu_4_ → AlCu) [25], as shown in Figure 2h and Figure 5e. When the peritectic phase (AlCu) completely covered the Al_3_Cu_4_, the peritectic reaction rate gradually decreased with the increase of AlCu layer thickness. Finally, the unconsumed Al_3_Cu_4_ is retained in the core of the AlCu, and the average thickness of AlCu is about 21 μm. Moreover, the AlCu (phase III_2_: J) can also be formed with a needle-like morphology through solidification (L → AlCu) when the temperature is lower than the peritectic reaction temperature (Figure 2h and Figure 5e,f). At the end of solidification, the remaining liquid phase converts to phase IV (F: Al_2_Cu) completely through peritectic reaction (L + AlCu → Al_2_Cu) and solidification (L → Al_2_Cu), as shown in Figure 2h and Figure 5f.

Based on the dynamic and static characterization, the final interfacial microstructure in Al/Cu samples from the Cu side to the Al side can be divided into layered solid diffusion microstructures (AlCu_3_, Al_4_Cu_9_, Al_2_Cu_3_ and AlCu) and solidification microstructures (Al_3_Cu_4_, AlCu and Al_2_Cu).

### 3.4. Growth Kinetics of IMCs

**Figure 7:**
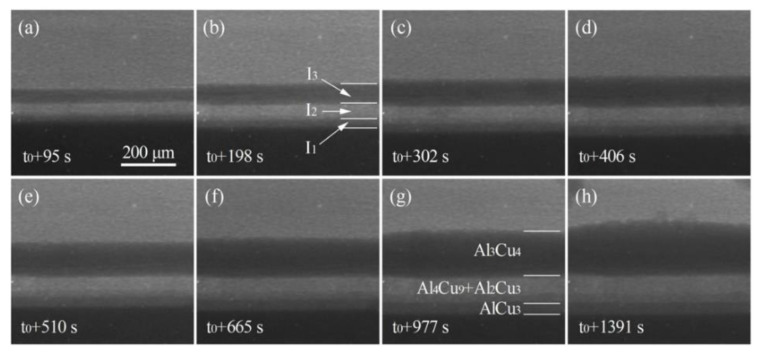
Sequence of in situ radiographs (**a**–**h**) showing the growth of IMCs layers (*t*_0_ is assigned to the onset of colling).

**Figure 8:**
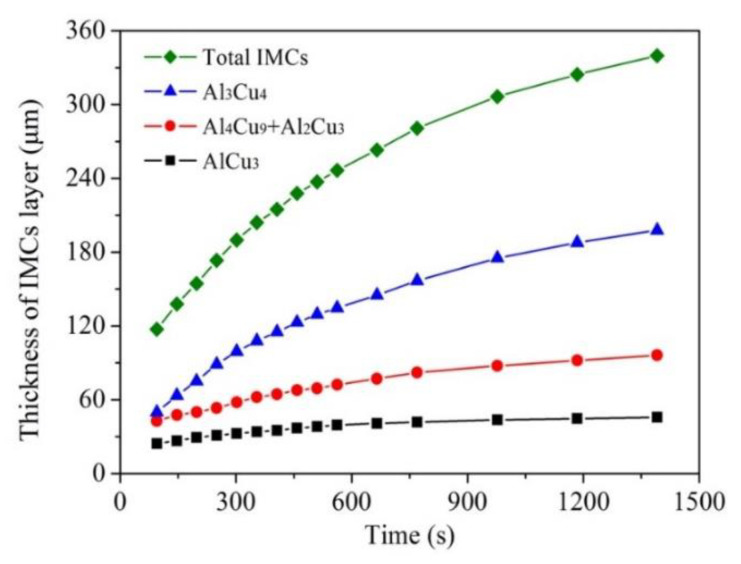
The time dependence of the thickness of IMCs layers.

Figure 7 shows the sequence of in situ radiographs of the growth of layered IMCs near the Cu side. For further quantitative study on the growth behavior of the IMCs, the growth curves of the IMCs were obtained from Figure 7 by image measurement, as shown in Figure 8. It can be seen from Figure 7 and Figure 8 that the thickness of all kinds of IMC layers increases with the increase in cooling time.

Generally, the relationship between the thickness of IMCs layer and the time can be expressed by an empirical power–law relationship [26,27]:

(3) $$x=x_{0}+k{\left(t\right)}^{n}$$

Taking the logarithm on both sides of Equation (3):

(4) ln Δx=ln k+n ln t

where, *x*_0_ and *x* are the thickness of the IMCs layer at time *t*_0_ and *t*, respectively. Δ*x* = *x* − *x*_0_, *k* is the growth rate constant, *n* is the time exponent.

In general, the growth mechanism of IMCs can be estimated by the value of *n* [28]. When *n* = 1, the growth is mainly controlled by the interfacial reaction, and the thickness has a linear relationship with diffusion time. When *n* = 0.5, the growth is mainly controlled by volume diffusion, and the thickness follows a linear relationship with the square root of diffusion time. When 0.5 < *n* < 1, the growth is controlled by both interfacial reaction and volume diffusion. 

**Figure 9:**
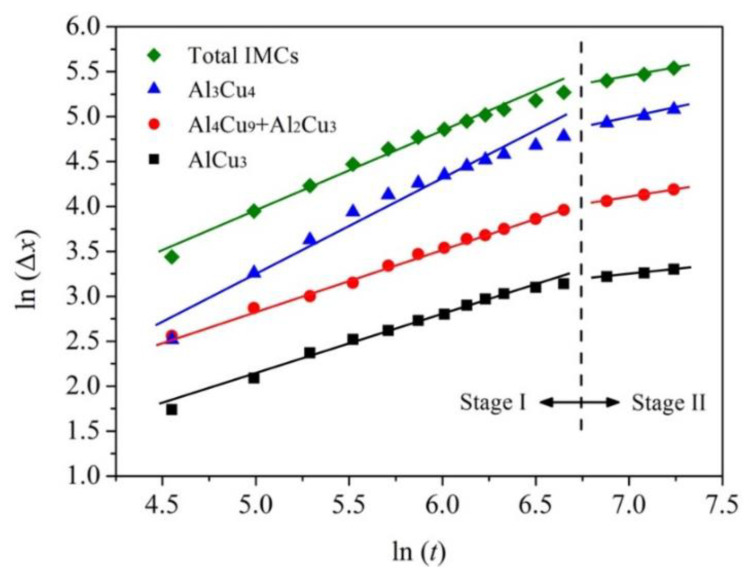
The relationship between *ln* (Δ*x*) and *ln* (*t*) for the IMCs layers.

According to Equation (4), the *n* values are obtained by plotting the logarithm of the IMC layer thickness (*ln*Δ*x*) and the logarithm of diffusion time (*lnt*), as shown in Figure 9 and Table 2. Based on the analysis of the curves’ slope, two different *n* values could be obtained by linear fitting for each IMC. That is, the growth of each IMC could be divided into two stages.

According to Table 2, the growth of AlCu_3_ and (Al_4_Cu_9_ + Al_2_Cu_3_) layers near the Cu side is all controlled by both interfacial reaction and volume diffusion in stage I. While the growth of the Al_3_Cu_4_ layer adjacent to the melt is mainly controlled by the interfacial reaction, and its growth rate is the highest. However, the *n* value corresponding to each IMC becomes smaller in stage II, which is mainly due to the lower temperature (at the end of solidification) in this stage and hinders the rapid diffusion of elements to a certain extent. Meanwhile, the gradual thickening of the compound layers also slows the elemental diffusion. Moreover, it is found that the *n* values of the Al_3_Cu_4_ and the total IMCs layers are relatively close in both stage I and II, indicating that the growth of Al_3_Cu_4_ plays a major role in the growth of the total IMCs.

## 4. Conclusions

The microstructural evolution and IMC formation at the liquid Al/solid Cu interface were studied using synchrotron radiation X-ray imaging technology combined with the static characterization method. The formation and types of interfacial microstructures are mainly affected by the degree of interdiffusion of elements in the Al/Cu samples, which can be divided into layered solid diffusion microstructures (AlCu_3_, Al_4_Cu_9_, Al_2_Cu_3_ and AlCu) and solidification microstructures (Al_3_Cu_4_, AlCu and Al_2_Cu) from the Cu side to the Al side. The formation, growth, collapse and floating of bubbles were observed during the melting, holding and solidification of the Al/Cu sample. Meanwhile, the growth of bubbles is mainly accomplished through diffusion, coalescence or engulfment. The thickness of IMCs increases with an increase in cooling time, and the growth of each IMC can be divided into two stages. The growth of AlCu_3_ and (Al_4_Cu_9_ + Al_2_Cu_3_) near the Cu side are all controlled by both interfacial reaction and volume diffusion. The growth of the Al_3_Cu_4_ adjacent to the melt is mainly controlled by the interfacial reaction, which plays a major role in the growth of the total IMCs.

## Figures and Tables

**Table 1:** Phases and morphology of the interfacial microstructure in Al/Cu sample.

SEM (Figure 5)	Phases	In-Situ Radiograph (Figure 2)	Morphology	Al-Cu Phase Diagram
A	AlCu_3_	Phase I_1_	Layered	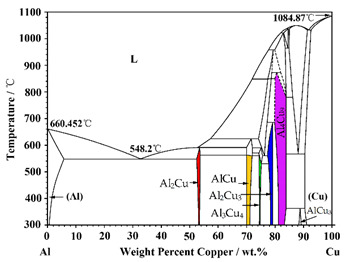
B	Al_4_Cu_9_	Phase I_2_	Layered	
C	Al_2_Cu_3_	Phase I_2_	Layered	
D	Al_3_Cu_4_	Phase I_3_	Layered	
H	Al_3_Cu_4_	Phase II	Petal-shaped	
E, G	AlCu	Phase III_1_	Cladding layer	
J	AlCu	Phase III_2_	Needle-like	
F	Al_2_Cu	Phase IV	—	

**Table 2:** The *n* values for IMCs layers at different stage.

Layer	AlCu_3_	Al_4_Cu_9_ + Al_2_Cu_3_	Al_3_Cu_4_	Total IMCs
Stage I	0.67	0.68	1.03	0.86
Stage II	0.22	0.36	0.42	0.39

## Data Availability

The data presented in this study are available on request from the corresponding authors.
